# Spatial statistical machine learning models to assess the relationship between development vulnerabilities and educational factors in children in Queensland, Australia

**DOI:** 10.1186/s12889-022-14541-7

**Published:** 2022-11-30

**Authors:** Wala Draidi Areed, Aiden Price, Kathryn Arnett, Kerrie Mengersen

**Affiliations:** 1 School of Mathematical Science, Center for Data Science, Queensland University of Technology, Queensland, Australia; 2 Children’s Health Queensland, Queensland, Australia

**Keywords:** Statistical machine learning methods, Spatial random forest, Deveplomental vulnerabilities

## Abstract

**Background:**

The health and development of children during their first year of full time school is known to impact their social, emotional, and academic capabilities throughout and beyond early education. Physical health, motor development, social and emotional well-being, learning styles, language and communication, cognitive skills, and general knowledge are all considered to be important aspects of a child’s health and development. It is important for many organisations and governmental agencies to continually improve their understanding of the factors which determine or influence development vulnerabilities among children. This article studies the relationships between development vulnerabilities and educational factors among children in Queensland, Australia.

**Methods:**

Spatial statistical machine learning models are reviewed and compared in the context of a study of geographic variation in the association between development vulnerabilities and attendance at preschool among children in Queensland, Australia. A new spatial random forest (SRF) model is suggested that can explain more of the spatial variation in data than other approaches.

**Results:**

In the case study, spatial models were shown to provide a better fit compared to models that ignored the spatial variation in the data. The SRF model was shown to be the only model which can explain all of the spatial variation in each of the development vulnerabilities considered in the case study. The spatial analysis revealed that the attendance at preschool factor has a strong influence on the physical health domain vulnerability and emotional maturity vulnerability among children in their first year of school.

**Conclusion:**

This study confirmed that it is important to take into account the spatial nature of data when fitting statistical machine learning models. A new spatial random forest model was introduced and was shown to explain more of the spatial variation and provide a better model fit in the case study of development vulnerabilities among children in Queensland. At small-area population level, increased attendance at preschool was strongly associated with reduced physical and emotional development vulnerabilities among children in their first year of school.

**Supplementary Information:**

The online version contains supplementary material available at 10.1186/s12889-022-14541-7.

## Introduction

Hospitals have started engaging their local populations in recent years to improve outreach and preventive health activities. Many of these efforts are being carried out under the name of enhancing “population health”. As Casalino and colleagues [1] stated, “Everyone in health care is working to improve population health these days. Or will be very soon. Or feel that they ought to be”. Hospitals which have typically focused on primary health care have started to acknowledge population health as a core component of their community commitment and strategic programs. Mutual service, health improvement, physical and environmental change and economic growth are supported through population health services [2].

Research conducted in 2017 by the American Hospital Association found that children’s hospitals invested a higher share of their overall community service costs than adult general hospitals [3]. Some children’s hospitals see population health as an opportunity for new initiatives to be adopted, to resolve the social determinants of health and to understand the need to shift current cultural institutional society to meet their objectives [1].

Participating in preschool programs the year before entering school has been reported to help children acquire healthy habits and can help to lessen disparities in development outcomes for vulnerable groups [4]. Preschool attendance has emerged as a national policy issue in many countries, including Australia. A variety of variables might impact whether or not a child attends preschool; for example, cultural obstacles to preschool participation might exist for non-English speaking and Indigenous households. Furthermore, the quality and quantity of preschool services available to children in rural and remote places may be less than in major cities [5].

**Fig. 1:**
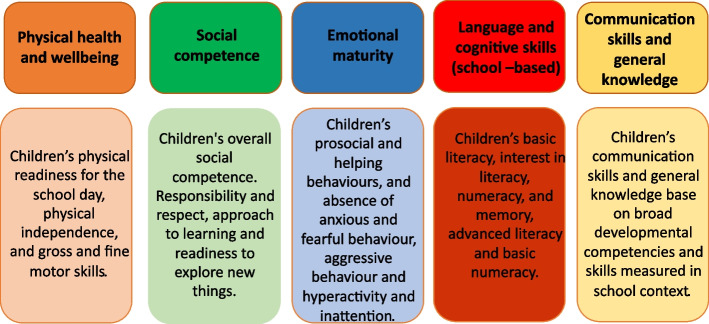
Five domains of children development from AEDC

The Australian Early Development Census (AEDC) [6] is a population-based cross-sectional census of early childhood development, derived from the Canadian Early Development Instrument. The AEDC elicits information about children’s demographics and early development outcomes (physical health and well-being, social competence, emotional maturity, language and cognitive skills (school-based), communication skills and general knowledge). Teachers complete the AEDC for all Australian children in their first year of compulsory school. Figure 1 shows the five domains of children development vulnerabilities measured by AEDC for children in their first year of school.

For reasons of privacy or communication, population health data and associated socio-demographic data collected about patients, families and constituent communities are often released at the level of small area aggregates. In Australia, these small areas are referred to by the Australian Bureau of Statistics (ABS) as statistical areas (SA1-SA4) [7]. The statistical areas are typically determined on the basis of health management or statistical divisions. It is common to practice to map these small statistical area data and assess demographic patterns in order to promote resource distribution and evidence-based policy making and planning. However, statistical analysis of aggregated spatial data presents specific challenges, particularly in assessing spatial patterns or identifying associations between health, potential socio-demographic factors and other potential explanatory variables. Using regression or classification approaches that ignore the spatial structure of data can be insufficient [8].

A range of statistical machine learning models are now available that take into account the spatial nature of the data. Simple approaches include adding geographic coordinates or distance metrics to familiar models such as linear regression, random forests and neural networks [9]. More sophisticated geographic extensions of these approaches, as well as combinations of models, have also been proposed [10–14].

Interestingly, we found that these spatial models may not capture all of the spatial autocorrelation in the data. The presence of spatial autocorrelation in the residuals after fitting a model suggests that the model estimates and predictions could be imprecise or biased [15]. In this article, we suggest a spatial random forest (SRF) model that can explain more of the spatial variation in the data than other common statistical machine learning approaches. We describe this approach in the context of a review of established popular aspatial and spatial statistical machine learning models, and compare the methods in a case study of development vulnerabilities among children in Queensland, Australia. The aims of the study are two fold: to evaluate spatial variation in these vulnerabilities, and to assess the relationship between the proportion of vulnerable children and the proportion of children attending preschool, based on aggregated small area data.

## Materials and methods

This section discusses the case study area and sources of data, then provides a short review of aspatial and spatial linear models, random forests and neural networks. A new spatial random forest method is also introduced in this section.

### Study area

Queensland is the second largest and third most populous Australian State or Territory, and is located in the northeast of the country. With strengths in mining, agriculture, tourism, international education, insurance, and banking. Queensland also has the third largest economy [16, 17]. The State is divided geographically into 528 non-overlapping statistical area level 2 (SA2) regions (according to the ASGS 2016 boundaries of the Australian Bureau of Statistics, ABS). SA2 regions are medium-sized general purposed areas that are designed to represent a community that interacts together socially and economically (www.abs.gov.au). This is the smallest area for the release of ABS non-census and inter-censal statistics, including the estimated resident population and health data, and data from the 2016 Census of Population and Housing.

In this study, health and socio-demographic data are obtained at the SA2 level for 526 SA2s, excluding those with zero population and with offshore/migratory or undefined location.

### The data repository

The outcome variables considered in this study were development vulnerabilities, provided by the Australian Early Development Census (AEDC). The AEDC takes place every three years and is the world’s most extensive data gathering for children. Classroom teachers complete the census for their students in their first year of full time school, and their answers are used to construct domain scores. Each child is given a score between zero and ten for each of the AEDC domains, using the cut-offs established as a baseline in 2009, children falling below the $10^{{th}}$ percentile in a domain, taking into account the age differences, are categorised as “developmentally vulnerable”. In Queensland, the percentage of children who are developmentally vulnerable in at least one domain in 2018 was around 26%, and the overall percentage of attendance at preschool was around 75.4% These are the lowest rates among all states and territories of Australia. There is also substantial geographic variation in rates across the state.

In this study, the outcome variable of interest is the SA2 level development vulnerability score for each domain, which is the age matched proportion of developmentally vulnerable children in the SA2. Five development vulnerabilities were considered in this study. These include: physical health and well-being domain vulnerability (PHD), social competence domain vulnerability (SCD), emotional maturity domain vulnerability (EMD), language and cognitive skills domain vulnerability (LCS), communication skills and general knowledge domain vulnerability (CS), and two development domain indicators which are vulnerable on one or more domain(VOD), and vulnerable on two or more domains (VTD).

The covariate information was extracted from the ABS and AEDC for each SA2. The covariates of interest obtained from the ABS included a geographic remoteness category, a Socio-Economic Index for Area (SEIFA) score, specifically an Index of Relative Socio-Economic Disadvantage (IRSD), mother’s language, country of birth, Indigenous status, and attendance at preschool. These covariates are also gathered as part of the survey AEDC and aggregated for research purposes.

The ABS classification of geographical remoteness is major city, inner-regional, outer-regional, remote and very remote. In Queensland there are 294 SA2 areas categorised as major cites, 113 SA2 areas as inner regional, 96 SA2 areas as outer regional, 11 SA2 areas as remote and 14 SA2 areas as very remote area [18].

The SEIFA score is a broad socioeconomic index that summarises a variety of data on individual and family economic and social condition in a given area. This factor is coded from 1 to 10. A low score suggests that the area in general is at a disadvantage. For example, low-income households, or people without qualifications or in low skill occupations.

Binary classifications were used for mother’s language (English, other), Indigenous status (Indigenous, not), Country of birth (Australia/not Australia) and attendance at preschool (yes, no).

The data custodians listed the above data over different time periods. In this study, we collect annual data only from 2018-2019. This study used the latest publicly available data from the 2018-2019 census. All count covariates acquired in this study have been transformed into proportions of children in an SA2 region with the feature of interest. Between 3% and 6% of the data were missing variables in the dataset. Missing continuous data was imputed using spatial neighbourhood averages. For categorical data, imputation was instead taken as the highest frequency neighbourhood category. In two instances, missing values for two islands could not be filled, as the regions have no contiguous neighbours. As a result, the analysis carried out in this study was reduced to the remaining 526 SA2 regions.

### Overall measures of spatial variation

Moran’s I [19] and Geary’s C [12] are popular measures to determine whether the data are geographically clustered, randomly distributed, or uniformly distributed in space. The semi-variogram, which depicts the range and rate at which spatial autocorrelation decreases, is another tool for measuring spatial dependency [20]. The semi-variance of a dataset with spatial autocorrelation typically grows to a maximum value before levelling off. The range of Moran’s I is between -1 and 1, where -1 is perfect dissimilarity clustering, 0 means that there is no spatial autocorrelation, and 1 indicates perfect similarity clustering.

Tangos’ maximized excess events test (MEET) [21] is another way to detect the spatial variation inside the data. This measure assumes a range of spatial scale parameters and depends on a weight function. Tango’s (MEET) has been shown to have very good statistical power in detecting global disease clustering [21]. Tango [22] proposed a distance based exponential weight function for MEET, but other choices of weights are also possible. one feature of this test is that it considers a range of spatial scale parameters, adjusting for the multiple testing Tango’s (MEET) has been shown to have very good statistical power in detecting global disease clustering. For more details see the Appendix.

### Statistical machine learning algorithms

#### Random forests for spatial data

A number of approaches have been proposed for applying a random forest to spatial data. Longitude and latitude were introduced as covariates in several efforts to integrate a spatial context into machine learning [13, 23, 24]. For example, Behrens [13] used x- and y-coordinates and distances to the corners and center of a bounding box around the sampling locations as covariates. Random Forest for Spatial Prediction (RFsp) was developed by Hengl [9], and uses buffer distance maps from observation points as covariates. In the next section we discuss another popular approach, the geographical random forest (GRF).

#### Geographical random forests

The GRF is a disaggregation consisting of several local sub-models [14]. It uses a similar idea to geographical weighted regression (GWR) [25]. Here, a local RF is computed for each location *i* based only on nearby observations. Thus for each training data point, a RF is developed, each with its own efficiency, predictive ability, and feature importance. As a result, the stability of the RF is measured locally rather than globally.

A GRF can be used to achieve two goals: firstly to enhance predictions over a standard RF, and secondly to extract spatially differentiated model parameter inferences. The degree of spatial variation in the data and the required bandwidth selection determine the increase in efficiency. Moreover, a GRF model can be used as a simple guide to investigate the data’s local structure and improve our understanding of how spatial processes affect this structure. For more details see the Appendix.

#### Neural networks for spatial data

One way of using neural networks for spatial data is to use the longitude and latitude as a covariate. We call this method a spatial neural network (SNN). Another recent extension of NN for spatial data is the geographically weighted artificial neural network (GWANN) [26]. Each output neuron of GWANN has as a geographic location associated to it. This allows the spatial distances between the observations and the output neuron’s location to be calculated. As a result, the connection weights between the hidden and output layers can be understood as a geographical weighted regression GWR model when estimated using a geographically weighted error function.

Garson [27] devised a method for calculating the relative importance of each of the input variables based on the connection weights. In this algorithm each variable’s input is stored as a weight in the network model, and the contribution of each of these variables to the output is largely determined by the magnitude and direction of these link weights. A positive connection weight enhances the magnitude of the network output, whereas a negative weight suppresses the value of the response variable [28]. For more details see the Appendix.

#### Linear models for spatial data

The generalized linear model (GLM) can be extended to include non-normal responses via a generalized linear model, or additive terms via generalised additive model GAM. A spatial GLM or a spatial GAM is another way to model the spatial data. Non-Gaussian error distributions and non-linear correlations between response and predictor variables are supported by these regression techniques.

In the most simple form, latitude and longitude can be used as model inputs [29].

The spatial autoregressive (SAR) model proposed by Whittle [30] is a spatial approach for describing the connection between dependent and independent variables by taking the spatial effect into account. It features an autoregressive structure that represents the spatial dependency of the attributes using a precision matrix that is generally a function of the proximity between regions [31]. Moran’s I [19] can be used to confirm the presence of spatial variation before the SAR model is used. Weights are used to indicate the impact of location effects on the data [32]. For more details see the Appendix.

#### Conditional Autoregressive Model (CAR)

Bayesian models are especially well adapted to spatial modelling because the information particular to each region may be represented as priors, and both correlated and uncorrelated spatial effects can be investigated [33]. For more details see the Appendix.

Non of the aforementioned algorithms can explain the spatial autocorrelation. Spatial autocorrelation in data can inflate bias in statistical analyses [15, 34, 35]. Failing to appropriately address this issue will likely lead to three major statistical problems. First, the standard errors might be underestimated. Consequently, that will make the regression model itself unreliable [36, 37]. Second, parameter estimates, such as the regression coefficients might be biased [38]. The inflation or deflation of predictors’ coefficients will induce the over or under-estimation, respectively, of their predictive power [39].

## Spatial random forest

In the GRF method [14], the authors introduced a local version of the RF algorithm for geographical data, where the RF ran locally for each location and its neighbourhood. The principal idea of GRF is similar to geographically weighted regression, in which they move to local computation rather than the global one. This means that a local RF is computed for each location but only includes a number of nearby observations. In this section we introduce an alternative to the GRF methods [14], based on an extension of the global random forest algorithm [40]. Here, a second stage is added to the RF to absorb residual spatial autocorrelation in the data. This algorithm is described as a set of three steps.

**Fig. 2:**
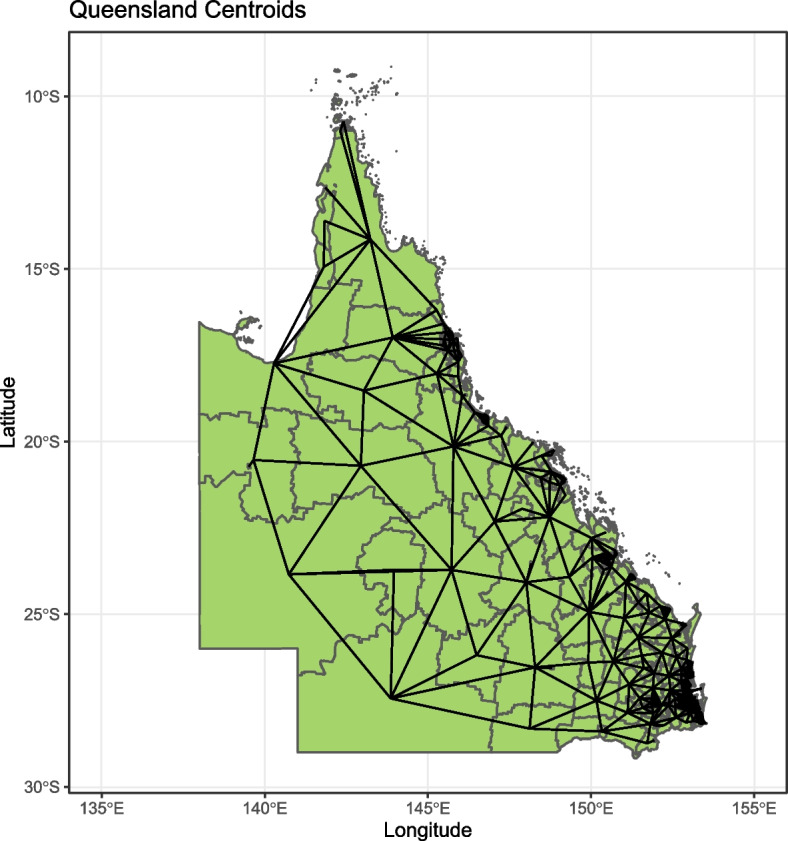
Queensland Centroid for SA2

- **Step 1**: Determine a neighbourhood for each spatial region. (In our case study we adopt a contiguous neighbour definition that accepts any region that shares at least one boundary). See Fig. 2.
- **Step 2**: Find the global random forest (RF):
  1 $$\begin{aligned} RF_1 \sim (y,x_i), \end{aligned}$$
- **Step 3**: Find the residual using the neighbourhoods
  2 $$\begin{aligned} r_i= \frac{\sum _{j \sim i} (y_j-\hat{y_j})}{n_j} \end{aligned}$$

Here, $y_i$ is the observed values, $\hat{y_i}$ is the estimated values using $RF_1$, and $j\sim i$ denotes all regions *j* in the neighbourhood of the *i*th region. Note that, in contrast to common measures such as mean absolute error (MAE) and mean square error (MSE), the neighbouring residuals are simply summed in the above equation. This is consistent with the concept of spatial correlation, in the set of residuals with different signs indicate a weaker spatial sign nature compared to a set with consistently positive region or negative signs.

- **Step 4**: Apply
  3 $$\begin{aligned} RF_2 \sim (y,\{x_i,r\}) \end{aligned}$$

Note that this method borrows conceptually from the conditional autoregression (CAR) approach. Our suggested algorithm, the spatial random forest (SRF) approach, better account for spatial auto-correlations by including an additional term representing the neighbourhood average of the difference between the estimated and observed response values. Our algorithm starts with running the RF algorithm and then computes and includes an additional term in a second run of the algorithm.

## Model evaluations

We use three well-established and reliable measures to assess model fit and accuracy: coefficient of determination $R^2$, root mean square error (RMSE) and Moran’s I. Here,

4 $$\begin{aligned} RMSE= \sqrt{\frac{1}{n}\sum \limits _{i=1}^n(x_{i}-y_{i})^2}, \end{aligned}$$

and

5 $$\begin{aligned} R^{2}= 1-\frac{\sum _{i=1}^n(y_{i}-\hat{y_{i}})^2}{\sum _{i=1}^n(y_{i}-\overline{y_{i}})^2}, \end{aligned}$$

where *n* is the total number of points, $y_{i}$, $\hat{y_{i}}$ and $\overline{y}_{i}$ are the actual values, estimated values, and the averaged values, respectively. Moran’s I [19] was discussed earlier and is another way to judge the consistency of a model applied to geographical and spatial data.

The importance of variables for the RF, SRF, GRF can be determined by the mean square error (iMSE) and impurity reduction. The impurity reduction introduced by a split is maximised using RF splitting criteria. A split with a significant decrease in impurity is considered important for the impurity. In addition, the impurity importance for a variable $x_i$ is calculated by the sum of all impurity decrease measures of all nodes in the forest. Consider splitting a regression tree *T* at a node *t*. Let *s* be a proposed split for a variable *X* that splits *t*. Regression node impurity is determined by within node sample variance

6 $$\begin{aligned} \delta (t) = {\frac{1}{N}\sum _{ x_i \in t}(Y_{i}-\overline{{Y_{t}}})^2}, \end{aligned}$$

where $\overline{{Y_{t}}}$ is the sample mean for *t* and *N* is the sample size of *t* [41].

## Case study analysis

For this case study, a number of data processing steps were required before the application of statistical models. First, relevant AEDC and ABS data were collected and converted to proportions at the SA2 level. This was achieved by dividing each region’s data by the population of children in their first year of school. To conduct adequate spatial analysis, the longitude, latitude, centroids, and contiguous boundaries were determined for each SA2 region and added to the data set. Figure 2 shows the contiguous centroids for each SA2 region in Queensland.

This data was then used inside statistical machine learning after splitting the data into training (80%) and testing (20%) sets. This division of data for training and testing is common in machine learning literature [42], with training data validated using 10-fold cross validation. A range of hyper-parameters were specified prior to model implementation, e.g., number of hidden layers, bandwidth, etc. See Appendix for more details.

The statistical analysis was conducted using the R programming environment [43–45] and utilised a number of packages, including Random Forest [46] for random forest calculations, ggplot2 [47] for visualizing the data, caret [48] for data preparation and separation, spatialML [49] for geographical random forest (GRF) model, neuralnet [50] for neural network and spatial neural network, GWANN [26] for geographical weighted artificial neural network and CARBayes [51] for Bayesian spatial linear regression modelling. In the random forest models analyses, the impurity reduction and the iMSE values were calculated using the testing data for each parameter to determine variable importance. The longitude and latitude were included as a covariates for spatial neural network and the relative importance was calculated. For the GAM model, cubic spline smoothing functions were used between the cut points. Cross validation was used to determine the optimal number of knots, and interactions between the covariates were also included in the model. After implementing the statistical machine learning methods, the values of $R^2$, *RMSE* and Moran’s I were calculated for each model.

## Results

**Fig. 3:**
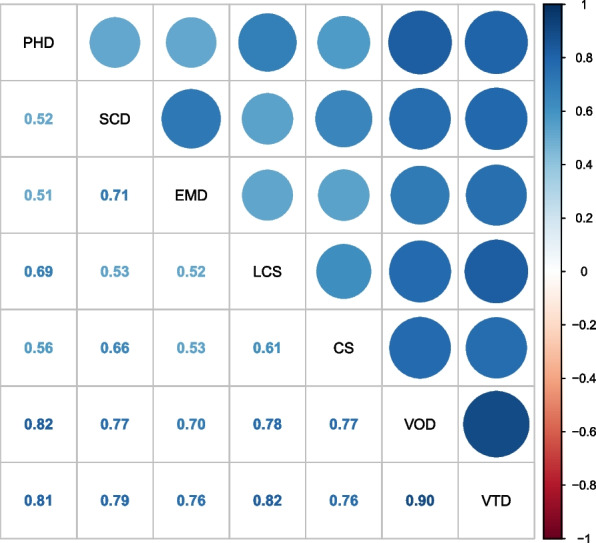
Correlation plot between different types of vulnerabilities

Figure 3 shows the correlation plot between the five domains of vulnerabilities and two indicators in the case study. The strongest correlation is between vulnerability on one or more domains (VOD) and vulnerability on two or more domains (VTD), where the Pearson correlation coefficient is 0.9. while the weakest correlation is between physical health domain vulnerability (PHD) and emotional maturity domain vulnerability (EMD) which is around 0.51.

Table 1 show the values of the coefficient of determination $R^2$ and the *RMSE* for the models considered in this study.

**Table 1:** Values of the $R^2$ and *RMSE* for different statistical machine learning models

Methods	PHD		SCD		EMD		LCS		CS		VOD		VTD	
	$R^2$	RMSE	$R^2$	RMSE	$R^2$	RMSE	$R^2$	RMSE	$R^2$	RMSE	$R^2$	RMSE	$R^2$	RMSE
GLM	0.299	0.091	0.212	0.082	0.166	0.096	0.385	0.082	0.372	0.063	0.417	0.093	0.411	0.061
SAR	0.336	0.042	0.224	0.040	0.214	0.034	0.555	0.033	0.382	0.036	0.429	0.049	0.426	0.041
RF	0.702	0.036	0.730	0.035	0.587	0.028	0.729	0.028	0.734	0.031	0.752	0.048	0.717	0.038
GRF	0.759	0.031	0.722	0.021	0.669	0.026	0.782	0.022	0.788	0.020	0.811	0.041	0.778	0.032
SRF	0.771	0.034	0.704	0.032	0.616	0.027	0.737	0.026	0.755	0.031	0.691	0.045	0.707	0.034
GAM	0.307	0.054	0.239	0.049	0.208	0.039	0.525	0.052	0.377	0.052	0.404	0.107	0.462	0.051
GAM with interaction	0.506	0.047	0.348	0.044	0.287	0.037	0.625	0.052	0.379	0.052	0.469	0.068	0.467	0.048
SGAM	0.559	0.042	0.472	0.052	0.323	0.037	0.623	0.038	0.42	0.038	0.614	0.066	0.623	0.044
NN	0.611	0.054	0.604	0.094	0.568	0.114	0.684	0.081	0.684	0.081	0.590	0.082	0.669	0.075
SNN	0.719	0.050	0.737	0.081	0.679	0.111	0.713	0.049	0.726	0.079	0.684	0.081	0.689	0.071
GWANN	0.694	0.054	0.705	0.50	0.662	0.038	0.707	0.047	0.737	0.047	0.671	0.067	0.691	0.052
CAR	0.879	0.031	0.729	0.034	0.787	0.026	0.875	0.290	0.771	0.029	0.802	0.056	0.826	0.034

*PHD* physical health and well-being domain, *SCD* social competence domain, *EMD* emotional maturity domain, *LCS* language and cognitive skills domain, *CS* communication skills and general knowledge domain, *VOD* vulnerable on one or more domain, *VTD* vulnerable on two or more domains, *GLM* generalized linear model, *SAR* spatial autoregressive model, *RF* random forest, *GRF* geographical random forest, *SRF* spatial random forest, *GAM* generalized additive model, *SGAM* patial generalized additive model, *NN* Neural network, *SNN* spatial neural network, *GWANN* geographical weighted artificial neural network, *CAR* conditional autoregression model

From these models we can see that the GAM with interaction performs better than the GAM without interactions, which indicates non linear and complex relationships between the socio-demographic and education covariates and the development vulnerabilities. This is reinforced by the improved fit of the RF and NN compared to the GAM and GLM models. The table also reveals that the value of including spatial information. The values of *RMSE* are reduced and the values of $R^2$ are increased considerably for SAR, GRF, RF, SGAM, GWANN and CAR models compared to their non-spatial counterparts.

Among the spatial models, the Bayesian CAR model provided the largest $R^2$ value, and this model and GRF gave the smallest *RMSE* values for most of health outcomes vulnerabilities.

The importance of attendance at preschool on the health outcomes vulnerabilities was assessed in the models that were considered to be reliable in term of goodness of fit $R^2$ and accuracy *RMSE*.

Table 2 shows the relative importance of attendance at preschool for the RF, GRF, and NN models. It can be seen that the attendance at preschool variable plays a major role in the analyses of the physical health and well being domain, and the emotional maturity domain in the RF, GRF and SRF models. In contrast, attendance at preschool does not appear to play a major role for vulnerability on one or more domain or two or more domains. Furthermore, Garson’s algorithm showed evidence that as attendance at preschool increased, the development vulnerabilities decreased, based on SA2 level data.

**Table 2:** The importance proportion form RF,GRF, SRF respectively, and the relative importance values for NN, for proportion of attendance at preschool (educational factor)

Responses	RF	GRF	SRF	SNN
PHD	0.28	0.46	0.33	-0.06
SCD	0.09	0.13	0.08	-0.02
EMD	0.39	0.45	0.24	-0.03
LCS	0.07	0.01	0.04	-0.07
CS	0.13	0.06	0.09	-0.01
VOD	0.03	0.04	0.02	-0.02
VTD	0.02	0.03	0.03	-0.03

**Fig. 4:**
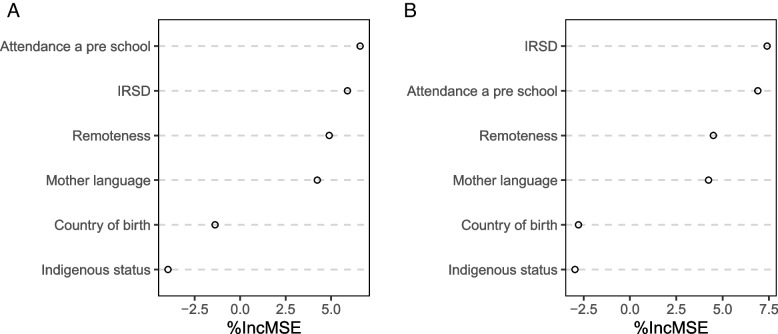
The importance plot using GRF for (**A**) physical health and well-being domain vulnerability (**B**) Emotional maturity domain vulnerability

Figure 4 show the values of the *iMSE* for the two vulnerabilities for which attendance at preschool was found to be important. It is apparent that attendance at preschool was the most important variable for physical health and wellbeing domain vulnerability, followed closely by IRSD, and second most important (after IRSD) for the emotional maturity domain vulnerability. These two variables, attendance at preschool and IRSD, were substantially more important than any of the other variables considered.

Table 3 shows the values of spatial autocorrelation (Moran’s I) for the residuals.

**Table 3:** Moran’s I (*P*-values) for the residuals from the different models

Responses	RF	GRF	SRF	SNN	GWANN	CAR
PHD	1e-04	1e-04	0.944	0.014	1e-04	0.001
SCD	0.012	0.002	0.942	0.0.231	0.001	0.796
EMD	1e-04	0.686	0.944	0.151	0.003	0.891
LCS	1e-04	0.008	0.944	0.0.027	1e-04	0.003
CS	0.104	0.0.002	0.942	0.489	0.003	0.003
VOD	1e-04	0.004	0.943	7e-04	0.005	0.004
VTD	0.003	0.021	0.943	0.024	0.004	0.101

According to this table the new spatial random forest was the only model to adequately fully explain spatial variation in the data, for all the health outcomes vulnerabilities. None of the GRF, GWANN or CAR models captured as much of the spatial variation.

Finally, Table 4 shows that the posterior median is substantively different from zero, since the credible interval does not include zero. The negative value indicates as the proportion of attendance at preschool increases the proportion of vulnerabilities between children decreases.

**Table 4:** The posterior medians and 95% credible intervals for the association between child development vulnerabilities and attendance at preschool parameter from different domains

Responses	Posterior median	95% Credible intervals
PHD	-0.071	[-0.139,0.001]
SCD	-0.071	[-0.134,-0.004]
EMD	-0.046	[-0.102, 0.012]
LCS	-0.058	[-0.115,-0.002]
CS	-0.081	[-0.141,-0.022]
VOD	-0.066	[-0.148, 0.018]
VTD	-0.093	[-0.168,-0.026]

The actual data and model estimates are presented as maps in the Supplementary material, for the SRF, GRF and CAR models.

## Discussion

This study analysed data from the 2018-2019 Australian early development census and Australian Bureau statistics, exploring five AEDC domains and two indicators across Queensland. For each domain and indicator, the proportion of vulnerable children in each small area (SA2) was mapped to highlight geographic variation and spatial patterns (see Appendix). The link between development vulnerabilities and educational factors such as attendance at preschool was also explored.

Despite government efforts to promote universal preschool attendance in Australia, the proportion of children attending preschool in Queensland is still the lowest in the nation. Universal access can ensure that a preschool program is available to all children whose parents desire them to attend, but it does not guarantee universal acceptance of this service [52]. Parental attitudes and preferences, child characteristics, and cultural norms are all variables that impact parents’ decisions to enrol their children in preschool [53]. Increasing preschool attendance can help all children achieve their developmental potential while also providing an opportunity to address disparities in developmental outcomes for children [54].

Consistent with previous studies [55–57], we found strong evidence that preschool attendance is inversely associated with child developmental vulnerability in the physical health domain. Preschool-age children are often perceived to be highly physically active, and previous studies have shown that 3- to 5-year-olds are more physically active than children in older age groups [57]. However, studies suggest that very young children are not as active as many believe [58]. Little is known about children’s physical activity levels while they attend preschools or how activity levels vary across preschools [59]. Our study found a high inverse association between attendance in preschool and vulnerability in the physical health domain.

Our findings also support the previous study on the association between attendance at preschool and the emotional vulnerability domain [60, 61]. The emotional domain suggests that the emotional goals of classrooms are consistent with and may even promote preliteracy skills. In other words, emotional goals are not at cross-purposes with academic goals and may help achieve them. Furthermore, researchers should continue efforts to create emotional assessment tools that educators may easily implement. With effective emotional assessment tools, educators may be better able to implement targeted interventions for specific emotional skill deficits. Broadening the focus of intervention efforts to include emotional skills increases the likelihood that every child’s need is academically and socially met [62, 63].

An increase in the number of children attending the preschool program and the amount of time spent in these settings. The number of physical activities children likely accumulate in preschool influences their health development.

It is also acknowledged that these findings should also be considered in light of other factors that may influence results, such as primary carer and parent education, weight at birth, single parent, and cultural sensitivity.

In addition, at the SA2 level, higher proportions of children associated with Aboriginal or Torres Strait Islander background, non-English background, remote areas and the relative socio-economic disadvantage were consistently associated with increased developmentally vulnerable in all domains. This highlights the strong influence of education and socio-economic and socio-demographic circumstances on early developmental capacities.

We conducted this study using statistical machine learning techniques to allow for the complexity of the interactions in the data. We also developed a new algorithm, the spatial random forest, which captures more of the spatial variation in the data. This is important to reduce bias and increase robustness of the results and corresponding inferences, and to help identify geographic variation. A comparison among different statistical machine learning algorithms was also conducted. The type of models included spatial and non spatial models. The traditional non spatial models showed poorer performance and accuracy than the spatial models, suggesting that the latter models are less biased and more robust in identifying important predictors related to child development. Among the spatial models, the drawback for the existing geographical random forest (GRF) model was that it needed more time to run in comparison with our spatial random forest (SRF) model and existing spatial neural network (SNN) models: GRF required around 6.25 minutes to run for each type of vulnerability with 400 bandwidths, while the SRF and NN needed 4.3 and 5.6 seconds, respectively. Bayesian spatial linear modelling needed 2.6 minutes to run. This result shows not only an improved statistical result but a faster computational run-time.

In this study, the spatial neighbourhood was defined based on shared boundaries. However, other options can be considered. For example, considering the average distances between neighbours for each region might work well to explain spatial autocorrelation for the random forest model.

The findings from this study offer important insights into both advancements in methodologies for applying statistical machine learning in public health and understanding child development in Queensland. However, we must consider the results in light of certain study limitations. First, although we assessed a wide range of non spatial and spatial model, it is acknowledged that other approaches may provide further insights into the case study. Moreover, further research is required to evaluate other variables and their interaction. Additional limitations relate to the reliance on survey data at a small area level of aggregation. Care must therefore be taken in making inferences at another level of aggregation or about individuals due to biases such as Simpson’s paradox [64] and the modifiable areal unit problem [65].

## Conclusion

The performance of different statistical machine learning algorithms and their corresponding predictions confirmed that it is crucial to consider the spatial nature of data when fitting a statistical machine learning model to analyse population health data at the SA2 level. A new spatial random forest model was introduced and was shown to explain more of the spatial variation and provide a better model fit than existing non-spatial and spatial models in the case study of development vulnerabilities among children in Queensland, Australia.

The study found increased associations between attendance at preschool and a range of development vulnerabilities, in particular, a strong inverse association with the physical health and emotional domains. These findings can help to inform early child health and education policies and facilitate more geographically targeted interventions.

## Supplementary Information

**Additional file 1:** **Appendix.**

## Data Availability

All the data used in this study are available to the public from the Australian Bureau of Statistic and Australian Early Development Census.
