# Supplementary material for: Spatial statistical machine learning models to assess the relationship between development vulnerabilities and educational factors in children in Queensland, Australia
Source: BMC Public Health. 2022 Nov 30;22:2232. doi: 10.1186/s12889-022-14541-7 (PMC9710117; doi:10.1186/s12889-022-14541-7)
Supplement: Supplementary file 1 — Additional file 1: Appendix. [file 12889_2022_14541_MOESM1_ESM.pdf]

A Overall Measures of Spatial Variation

Moran's I

Moran's I [18] can be calculated as

$$I = \frac{n \sum_{i=1}^{n} \sum_{j=1}^{n} w_{i,j} z_i z_j}{S \sum_{i=1}^{n} z_i^2}, \tag{1}$$

where $z_i = x_i - \overline{x}$ and $S = \sum_{i=1}^{n} \sum_{j=1}^{n} w_{i,j}$. Here, x_i is the independent variable, $\overline{x}$ is the associated sample mean and w_{ij} is an element of the spatial S matrix, which shows the degree of spatial connection between regions i and j. There are two types of the spatial weight matrix [17]. One type is based on distances among observations (e.g., inverse distances raised to some power, bandwidth as the nth nearest neighbour distance, ranked distances, spatial kernel functions), and the second type is calculated using contiguity relationships. since nearest neighbours typically supply enough spatial dependence information in spatial modelling and prediction [9]. As for individual-tree data, Voronoi cells are one of the optimal ways of identifying trees' nearest neighbours. In a contiguity matrix, a value of 0 indicates no common border between two observations, and 1 indicates the presence of a common border between observations which we use in the formula. [14]

Tango's MEET

Let c_i be the observation in a region i, n_i the population size of the region i, C the total number of observations, N the total population, d_{ij} the distance between region i and j, and $u_{j(i)}$ the population size in region i and its j nearest neighbors. then Tango's EET can be defined as:

$$\text{Tango's EET} = \sum_i \sum_j w_{ij}(c_i - n_i \frac{C}{N})(c_j - n_j \frac{C}{N}) \tag{2}$$

where w_{ij} is typically defined as $w_{ij} = e^{-4(\frac{d_{ij}}{\lambda})^2}$ [26] or $w_{ij} = e^{(-\frac{d_{ij}}{\lambda})}$ [28] where, λ is a measure of spatial scale clustering. Another weighted function based on the nearest neighbour is:$w_{ij} = (1/l)^s$ where l indicates the closest area to area i. A second choice is the adjacent neighbourhood weight which takes the value 1 if area i and j are neighbours or $i = j$ and 0 otherwise. Finally, a population-based weight is another possible weight function which uses the product of the proportion of the corresponding counties to the total population and is given as: $w_{ij} = \frac{n_i}{N} * \frac{n_j}{N}$ [26, 28].

B Statistical machine learning algorithms

Random Forest

A popular type of machine learning method is the decision tree, which has been shown to be fast, flexible, and can deal with large amounts of data [27]. A decision tree is built by grouping data using a recursive binary partitioning algorithms into more homogeneous groups. Each binary split is selected based on specified splitting criteria.

The random forest (RF) approach suggested by Breiman [5] creates and combines a large number of individual decision trees generated from the data set of interest. Random forests address some of the drawbacks of having a single classification and regression tree (CART), such as over-fitting, correlation between variables and trees sets of splits to describe non linear relationships. It is a combination of random sub-space methods [22] and bagging [3]. Every decision tree is randomly generated by sampling a proportion(usually approximately two-thirds) of the training data and leaving the reminder out of

training. Furthermore, at each decision node, only a subset of features is picked at random during the tree construction process. The final result is generated using the majority vote (classification) or the average prediction of all trees (regression). Simultaneously, the data left out of training for each tree is used to compute an output goodness of fit assessment called the out-of-bag (OOB) error estimate [5]. The importance of the covariates can be calculated using the OOB error, where the most effective approach is to use the increase in the mean squared error (iMSE) measure to determine variable importance. The values of each feature are permuted randomly, and the OOB error iMSE (increase in mean square error) is calculated . This method can be used for both regression and classification problems in many different fields [29, 20, 1, 21, 2].

Spatial decision tree

Breimanet [5] presented CART (Classification And Regression Trees) as a statistical approach for building tree predictors for regression and classification. In the classification instance, each observation is defined by input variables collected in vector X and a binary label Y that serves as the output (or response) variable. The general principle of CART is to recursively partition the input space using binary splits and then determine an optimal partition for prediction. The traditional representation of the model connecting Y to X is a tree that represents the model's underlying creation process. If the explanatory variables are spatial coordinates, we obtain a spatial decision tree, which causes the space to tessellate (of the X variables). A cell of this tessellation corresponds to a leaf of the decision tree. For a leaf of this tree, the response variable Y is constant and corresponds to the majority label of the observations belonging to this leaf. In the spatial database approach, classification is viewed as an organisation of items using their characteristics and their neighbours' properties. These might be direct neighbours, neighbours of neighbours, and so on, up to degree N. The presence of geographic variables in data differentiates a decision tree from a spatial decision tree, but the phases are the same. A geographical decision tree, like entropy in a decision tree, is used to assess a sample data set's heterogeneity (diversity). Entropy increases as the data set becomes more varied [12, 30].

Geographical Random forest

To understand more about this algorithm, we use a regression equation of the form,

$$Y_i = ax_i + e, \quad i = 1 : n \tag{3}$$

where Y_i is the ith observation's value of the response variable, ax_i is the RF's nonlinear prediction based on a set of x covariates, and e is the error term. For GRF, equation (3) is extended as follows.

$$Y_i = a(u_i, v_i)x_i + e, \quad i = 1 : n; \tag{4}$$

where $a(u_i, v_i)x_i$ is the RF model prediction for location i. and (u_i, v_i) are the spatial coordinates. The neighbourhood (or kernel) is the field in which the sub-model runs. For each location, a sub-model is created takes into account just nearby observations [9]. The area that the sub model operates in is called the neighbourhood (or kernel), and the maximum distance between a location i and its neighbourhood is called the bandwidth [15]. The neighbourhood is defined by n nearest neighbours or by a circle whose radius is the bandwidth (number of the nearest neighbours). Neighbours are defined as spatial units which share an edge or vertex [24]. The bandwidth is the maximum distance between a data point and its kernel [6]. There are two kinds of kernels that are commonly used, "adaptive" and "fixed" [13]. When sampling density varies across space, using an adaptive kernel is beneficial [9].

Neural Network

Like the RF, Neural Networks (NN) provide more representational flexibility and freedom from the constraints of a linear model [25]. A NN is made up of many or perhaps millions of tightly linked basic processing nodes. The majority of today's neural networks are structured into layers of nodes and are "feed-forward," meaning that data flows in just one direction through them. A single node may be linked to multiple nodes in the layer below it from which it receives data, as well as several nodes in the layer above it from which it transmits data. A node will assign a numerical "weight" to each of its incoming connections. Each node receives a new data item a distinct number, and multiplies it by the associated weight. The resulting items are then added together to produce a single number. If that number falls below a certain threshold, the node does not send any data to the next layer.
An input, hidden, and output layer make up a basic NN. The connection weights of a basic NN from the hidden to the output layer can be interpreted as the coefficients of a linear model of non-linearly transformed variables, namely the outputs of the hidden neurons [10].

Neural Network for Spatial Data

The key distinction between a GWANN and a basic ANN is that a GWANN calculates an error signal using a geographical weighted error function rather than the standard quadratic error function [10] [7]. The geographically weighted error function is given by:

$$E = \frac{1}{2}\sum_{i=1}^{n} v_i(t_i - o_i)^2, \tag{5}$$

where t_i is the target value, o_i the output of output neuron i, v_i the geographically weighted distance between the observation and the location of output neuron i, and n the number of targets. From equation (5), the difference between the output neuron's value and the target value is weighted by the spatial distance between the output neuron's location and the observation; when the output neuron's location and observation are close, the difference is given more weight than when they are farther apart.
A 10-fold cross-validation (CV) is typically used to calculate the number of GWANN training iterations. The models are trained within each fold until their performance on the current fold's test data does not increase after many iterations. The additional iterations are designed to offer networks a chance to break free from local minima. This method, known as "early stopping with patience" [4] minimises the chance of overfitting the training data significantly. The iteration with the best mean performance across all folds, as well as the performance value obtained, are then presented.

Relative importance

Garson [8] devised a method for calculating the relative importance of each of the input variables based on the connection weights. In this algorithm, each variable's input is stored as a weight in the network model, and the contribution of each of these variables to the output is largely determined by the magnitude and direction of these link weights. A positive connection weight enhances the magnitude of the network output, whereas a negative weight suppresses the value of the response variable [19]. Furthermore, when compared to the other factors, a variable with a considerably larger connection weight is regarded to have a bigger influence on the network output. Thus,

$$RI_x = \sum_{y=1}^{m} w_{xy}w_{yz} \tag{6}$$

where RI_x denotes the relative importance of input neuron x, w_{xy} the final weights of the connection from input neuron to hidden neurons, w_{yz} the final weights of the connection from hidden neuron to output neuron. y represents the total number of hidden neurons, and z is the output neuron.

Linear Model

Given a dependent or response vector y, and a matrix of input variables X, a general linear model can be expressed as

$$Y = X\beta + \epsilon \tag{7}$$

where β is a vector of regression coefficients and ϵ is a vector of residuals. In normal linear regression, ϵ is assumed to have a zero mean Gaussian distribution. The matrix X contains independent variables or covariates $\{X_1, X_2, ..., X_p\}$, as well as any interactions or functions of these variables (e.g, quadratic or cubic terms to allow for non-linear relationships between the independent and response variables).

Linear Models for Spatial Data

generalized linear models (GAMs) are non-parametric extensions of linear model regressions in which non-parametric smoothers f are applied to each predictor, and the component response is calculated additively, i.e.,

$$E(Y) = \beta_0 + f_1(x_1) + f_2(x_2) + f_3(x_3, x_4) + ... + f_k(x_k). \tag{8}$$

spatial regression

The general formula of the spatial regression called SARMA (Spatial Autoregressive Moving Average [11]) is given as

$$Y = \rho WY + X\beta + \lambda Wu + \epsilon \tag{9}$$

where Y is the dependent variable, X is the matrix of independent variables, ρ is the spatial autoregressive parameter, W is a weights matrix, β is a regression coefficient vector, λ is a spatial error coefficient, and ϵ is a residual vector.

Conditional Autoregressive Model (CAR)

A popular spatial prior proposed by Leroux [16] includes a spatial random effect ψ such that,

$$\psi \sim MVN(0,\, D) \tag{10}$$

with covariance matrix D, where D is usually described by its generalized inverse [16]

$$\sigma^2 D^- = (1 - \rho) + \rho R. \tag{11}$$

Here, R is the intrinsic autoregression matrix which represents the neighbourhood structure of the regions with typical element R_{ij}, which equals n_i when $i = j$, where n_i is the numbers of neighbours of region i, and $I(i \sim j)$ otherwise, where $I(i \sim j)$ is an indicator function taking the value 1 when i and j are neighbours.

The term ρ is introduced as a spatial dependence parameter, $\rho \in [0, 1]$, whose two extreme cases give rise to the independence model (i.e., $\psi_i = v_i$ and $D = \sigma^2 I$). The spatial residual is typically considered to have an independent normal distribution $v_i \sim \mathcal{N}(0, \sigma_v)$, and intrinsic auto regression (i.e., $\psi_i = u_i$ and $D = \sigma^2 R^-$), respectively [16]. For ρ close to 1, the conditional variance becomes close to σ^2/n_i and for ρ close to 0, the variance becomes close to σ^2, that is independent of the number

of neighbours n_i [23].
The univariate full conditional distribution for $\psi_i|\psi_{-i}$ can be written as

$$\psi|\psi_{-i} \sim \mathcal{N}(\frac{\rho}{n_i\rho + 1 - \rho}\sum_{j\sim i}\psi_j, \frac{\sigma^2}{n_i\rho + 1 - \rho}) \tag{12}$$

where ψ_{-i} denotes the random effect vector with the ith component deleted.

C Hyper-parameters of the models used for the case study

Random Forest

The most common parameters for the random forest are mtry and ntree, which have been found using 10-fold cross-validation and three repeats (random search) in caret packages. We found mtry = 3, and to find the ntree, we tried different values (500,1000,1500,2000,2500) and calculated the RMSE. The optimal number of trees was 1000.

Spatial Random Forest

The same method has been used to find the optimal parameters. We found mtry=3, ntree=1000

Geographical Random Forest

For geographical random forest, we used the adaptive kernel, which can be used when the density is different across space – which is the case in our dataset, the bandwidth setup to be 400, which gave the lowest RMSE. mtry=3 , ntree=1000

Neural network

hidden=c(10,3), threshold=0.01, stepmax = 100,000

Spatial Neural Network

Hidden=c(10,3), threshold=0.01, stepmax = 100,000, we added longitude and latitude as a covariate in this model

Geographical Weighted Artificial Neural Network

learning rate=0.01,batchSize=50,nrHidden=10, adaptive=F, cv patience=999,cv max iterations=99999,cv=10, bwSearch=”goldenSection” ,bandwidth=NA, threads=8 (Bandwidth NA to determinate using cross-validation,bwSearch Method for searching an appropriate bandwidth (golden section or grid).

Conditional Autoregressive Model

nsample=30000, burnin=10000,thin=100

D Spatial maps

The actual and estimated responses using Spatial Random Forest (SRF), Geographical Random Forest (GRF), and CAR model. Where A: the actual responses, B: the estimated responses from SRF, C: the estimated responses from GRF, D: the estimated responses from CAR model

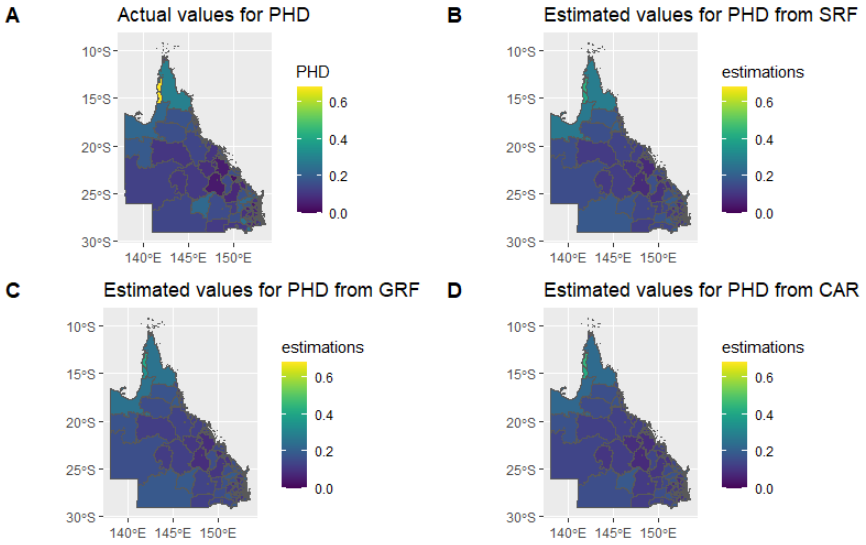

Figure 1: Physical health and wellbeing domain vulnerability.

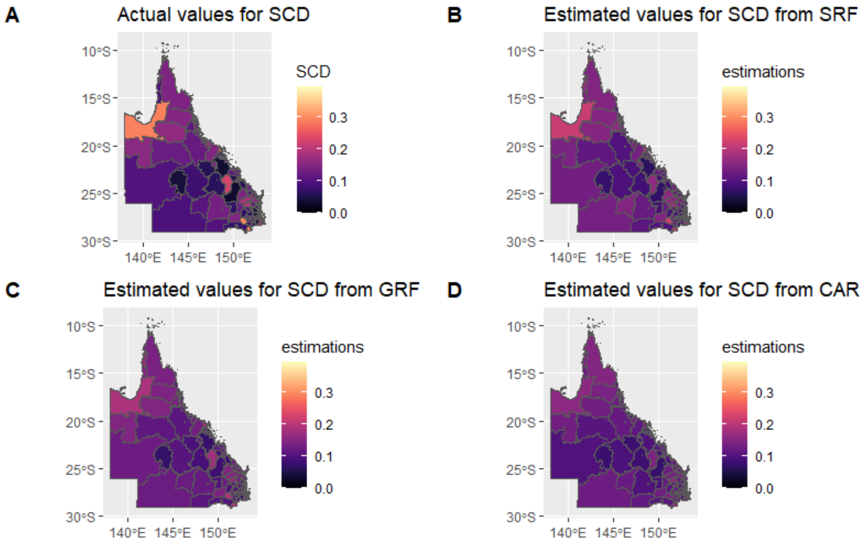

Figure 2: Social competence domain vulnerability.

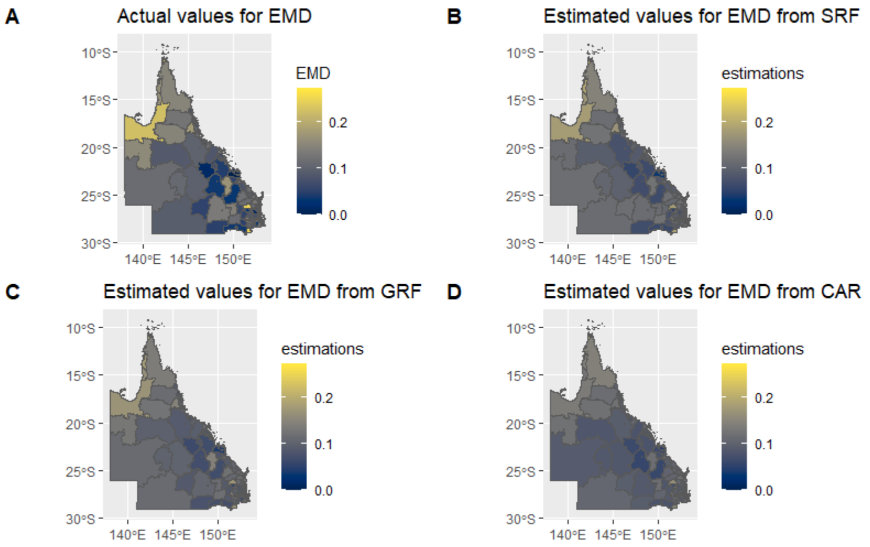

Figure 3: Emotional maturity domain vulnerability.

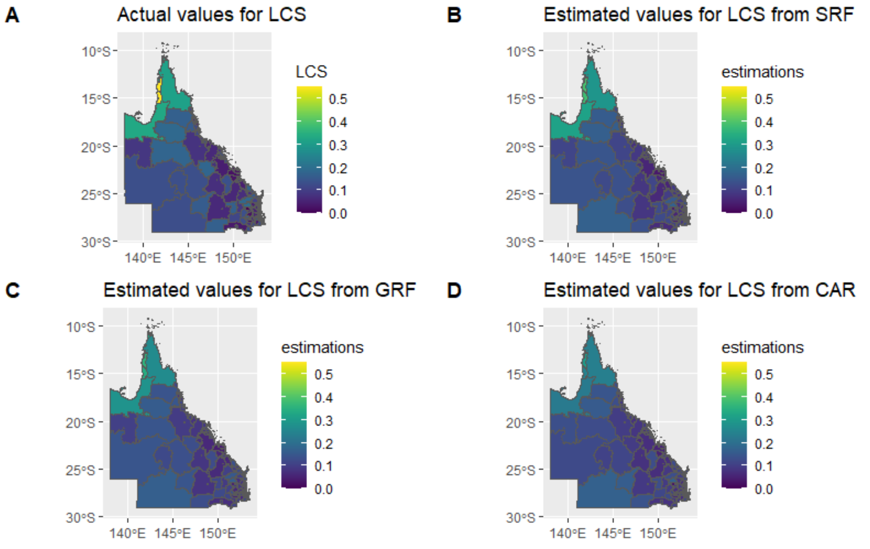

Figure 4: Language and cognitive skills domain vulnerability.

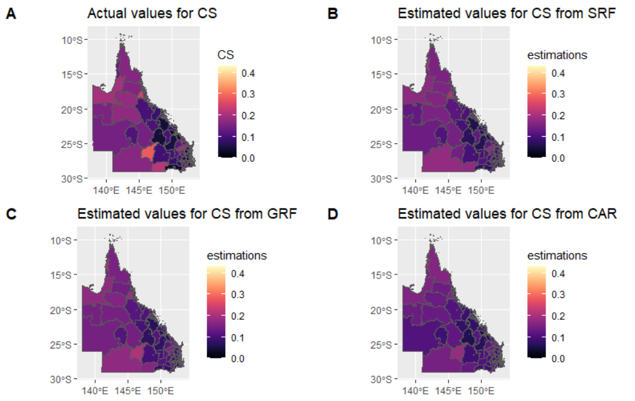

Figure 5: Communication skills domain vulnerability.

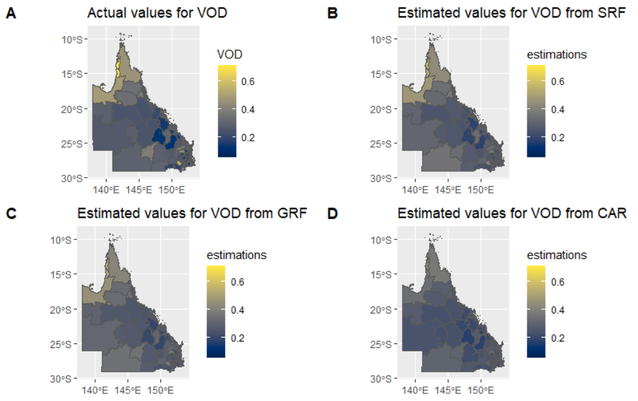

Figure 6: Vulnerability on one or more domain/domains vulnerability.

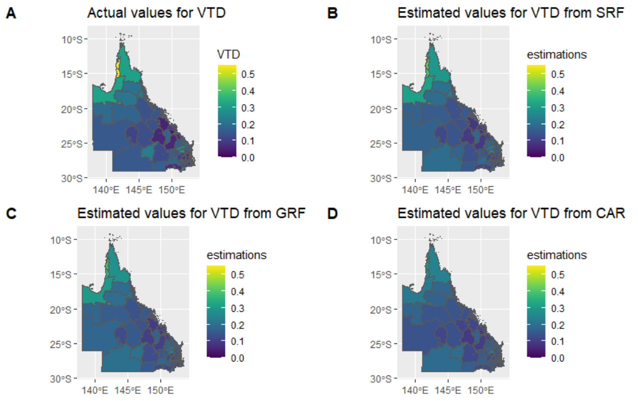

Figure 7: Vulnerability on two or more domains vulnerability.

References

[1] J. Albert, E. Aliu, H. Anderhub, P. Antoranz, A. Armada, M. Asensio, C. Baixeras, J. Barrio, H. Bartko, D. Bastieri, et al. Implementation of the random forest method for the imaging atmospheric cherenkov telescope magic. *Nuclear Instruments and Methods in Physics Research Section A: Accelerators, Spectrometers, Detectors and Associated Equipment*, 588(3):424–432, 2008.

[2] Y. Ao, H. Li, L. Zhu, S. Ali, and Z. Yang. The linear random forest algorithm and its advantages

in machine learning assisted logging regression modeling. *Journal of Petroleum Science and Engineering*, 174:776–789, 2019.

[3] A. Arfiani and Z. Rustam. Ovarian cancer data classification using bagging and random forest. In *AIP Conference Proceedings*, volume 2168, page 20046. AIP Publishing LLC, 2019.

[4] Y. Bengio. Practical recommendations for gradient-based training of deep architectures. *Neural networks: Tricks of the trade*, pages 437–478, 2012.

[5] L. Breiman. Random forests. *Machine Learning*, 45(1):5–32, 2001.

[6] C. Brunsdon, S. Fotheringham, and M. Charlton. Geographically weighted regression. *Journal of the Royal Statistical Society: Series D (The Statistician)*, 47(3):431–443, 1998.

[7] Z. Du, Z. Wang, S. Wu, F. Zhang, and R. Liu. Geographically neural network weighted regression for the accurate estimation of spatial non-stationarity. *International Journal of Geographical Information Science*, 34(7):1353–1377, 2020.

[8] D. Garson. Interpreting neural network connection weights. *Computer Science*, 1991.

[9] S. Georganos, T. Grippa, A. Niang Gadiaga, C. Linard, M. Lennert, S. Vanhuysse, N. Mboga, E. Wolff, and S. Kalogirou. Geographical random forests: a spatial extension of the random forest algorithm to address spatial heterogeneity in remote sensing and population modelling. *Geocarto International*, pages 1–16, 2019.

[10] J. Hagenauer and M. Helbich. A geographically weighted artificial neural network. *International Journal of Geographical Information Science*, pages 1–21, 2021.

[11] J. Huang. The autoregressive moving average model for spatial analysis. *Australian Journal of Statistics*, 26(2):169–178, 1984.

[12] Z. Jiang, S. Shekhar, P. Mohan, J. Knight, and J. Corcoran. Learning spatial decision tree for geographical classification: a summary of results. In *Proceedings of the 20th International Conference on Advances in Geographic Information Systems*, pages 390–393, 2012.

[13] S. Kalogirou. Destination choice of Athenians: an application of geographically weighted versions of standard and zero inflated poisson spatial interaction models. *Geographical Analysis*, 48(2):191–230, 2016.

[14] S. Kalogirou and T. Hatzichristos. A spatial modelling framework for income estimation. *Spatial Economic Analysis*, 2(3):297–316, 2007.

[15] T. Koç. Bandwidth selection in geographically weighted regression models via information complexity criteria. *Journal of Mathematics*, 2022, 2022.

[16] B. Leroux, X. Lei, and N. Breslow. Estimation of disease rates in small areas: a new mixed model for spatial dependence. *Statistical Models in Epidemiology, the Environment, and Clinical Trials*, pages 179–191, 2000.

[17] Q. Meng, C. J. Cieszewski, M. R. Strub, and B. E. Borders. Spatial regression modeling of tree height–diameter relationships. *Canadian journal of forest research*, 39(12):2283–2293, 2009.

[18] P. Moran. The interpretation of statistical maps. *Journal of the Royal Statistical Society: Series B (Methodological)*, 10(2):243–251, 1948.

[19] J. Olden and D. Jackson. Illuminating the “black box”: a randomization approach for understanding variable contributions in artificial neural networks. *Ecological modelling*, 154(1-2):135–150,

2002.

[20] S. Oliveira, F. Oehler, J. San-Miguel-Ayanz, A. Camia, and J. M. Pereira. Modeling spatial patterns of fire occurrence in mediterranean Europe using multiple regression and random forest. *Forest Ecology and Management*, 275:117–129, 2012.

[21] M. Pal. Random forest classifier for remote sensing classification. *International Journal of Remote Sensing*, 26(1):217–222, 2005.

[22] P. Panov and S. Džeroski. Combining bagging and random subspaces to create better ensembles. In *International Symposium on Intelligent Data Analysis*, pages 118–129. Springer, 2007.

[23] R. Rampaso, A. de Souza, and E. Flores. Bayesian analysis of spatial data using different variance and neighbourhood structures. *Journal of Statistical Computation and Simulation*, 86(3):535–552, 2016.

[24] A. Sekulić, M. Kilibarda, G. Heuvelink, M. Nikolić, and B. Bajat. Random forest spatial interpolation. *Remote Sensing*, 12(10):1687, 2020.

[25] H. Sharma, J. Park, D. Mahajan, E. Amaro, J. K. Kim, C. Shao, A. Mishra, and H. Esmaeilzadeh. From high-level deep neural models to FPGAs. In *2016 49th Annual IEEE/ACM International Symposium on Microarchitecture (MICRO)*, pages 1–12. IEEE, 2016.

[26] C. Song and M. Kulldorff. Tango's maximized excess events test with different weights. *International Journal of Health Geographics*, 4(1):1–7, 2005.

[27] Y. Song and L. Ying. Decision tree methods: applications for classification and prediction. *Shanghai Archives of Psychiatry*, 27(2):130, 2015.

[28] T. Tango. A class of tests for detecting 'general' and 'focused' clustering of rare diseases. *Statistics in Medicine*, 14(21-22):2323–2334, 1995.

[29] P. Zahedi, S. Parvandeh, A. Asgharpour, B. McLaury, S. Shirazi, and B. McKinney. Random forest regression prediction of solid particle erosion in elbows. *Powder Technology*, 338:983–992, 2018.

[30] M. Zhao and X. Li. An application of spatial decision tree for classification of air pollution index. In *2011 19th International Conference on Geoinformatics*, pages 1–6. IEEE, 2011.
